# Psychological wellbeing of Ukrainian civilians: a data report on the impact of traumatic events on mental health

**DOI:** 10.3389/fpsyg.2025.1553555

**Published:** 2025-06-26

**Authors:** Anton Kurapov, Oleksandra Balashevych, Yelyzaveta Borodko, Yelyzaveta Vovk, Anastasiia Borozenets, Ivan Danyliuk

**Affiliations:** ^1^Department of Experimental and Applied Psychology, Faculty of Psychology, Taras Shevchenko National University of Kyiv, Kyiv, Ukraine; ^2^Department of Psychology, Centre for Cognitive Neuroscience, Faculty of Natural Sciences, University of Salzburg, Salzburg, Austria; ^3^Department of Psychodiagnostics and Clinical Psychology, Faculty of Psychology, Taras Shevchenko National University of Kyiv, Kyiv, Ukraine

**Keywords:** war, Ukraine, PTSD symptoms, sleep quality (SQ), mental health, wellbeing

## Introduction

The Russian-Ukrainian war has profoundly impacted the lives of Ukrainians, bringing both direct and indirect psychological challenges. Armed conflicts, such as this one, are associated with enduring effects on mental health, including post-traumatic stress disorder (PTSD), anxiety, depression, and psychological distress, which affect all demographic groups irrespective of their financial status, marital status, education, or gender (Chudzicka-Czupała et al., 2023; Garry and Checchi, 2020). However, the degree of vulnerability varies, with those exposed to war-related events being at the highest risk.

Direct exposure to war, including constant shelling, loss of homes, displacement, and violence, has left a significant mark on mental wellbeing. Many civilians face daily threats to their lives and are dealing with injuries, deaths of loved ones, and destruction of infrastructure. The closer to the frontline regions where people often lack access to necessities such as food, water, electricity, heating, and shelter, the higher the level of stress and anxiety is reported because people are facing the following difficulties more often (Pypenko et al., 2023). These daily stressors, worsened by the armed conflict, compound existing challenges, making mental recovery and resilience harder to achieve (Miller and Rasmussen, 2010; Palace et al., 2024). Another threat for young adults in Ukraine is a substantial risk of screening positive for depression, anxiety, insomnia, and PTSD symptoms due to their exposure to violent events like shellings, explosions, occupation, violence, assault, and witnessing or experiencing the death of loved ones (Polyvianaia et al., 2025). The stated above symptoms often co-occur simultaneously.

The economic ramifications of the war further exacerbate mental health struggles. Rising energy and commodity prices, along with rise in food prices, have increased the economic burden on civilians already affected by war (Balbaa et al., 2022). This has contributed to financial hardship for a lot of Ukrainians. Along with financial difficulties, factors such as social marginalization, isolation, inadequate housing conditions, and changes in family structure and functioning can trigger or intensify a range of severe stressors (Miller and Rasmussen, 2010). Thus, the socio-economic consequences of the war are also likely to worsen the psychological wellbeing of Ukrainians.

According to the research, only 23.5–26% of civilians exposed to traumatic events were diagnosed with PTSD (Ahmed et al., 2024; Lim et al., 2022; Morina et al., 2018). However, traumatic events can still significantly impact the mental health of the broad population of civilians. Despite the generally high levels of resilience observed in populations, those directly exposed to military actions, violence, or severe human suffering continue to experience significantly elevated levels of stress, anxiety, and trauma-related symptoms (Kurapov et al., 2023). The report follows an initial screening conducted by our research team (Kurapov et al., 2023) and represents one of a planned series of assessments. This study serves as a checkpoint for monitoring the mental health of Ukrainians during the ongoing war, providing a valuable tool for tracking changes and dynamics over time. It offers a screening of mental health among Ukrainian civilians, an overview of key dimensions such as sleep quality, anxiety, emotional wellbeing, PTSD symptoms, alcohol consumption, and the long-term effects of traumatic events. The findings aim to present an overview of trends in the psychological state of Ukrainians. This report may be used to guide the development of evidence-based programs for Ukrainian civilians and inform mental healthcare systems, psychologists, and international partners addressing the consequences of this war in one of today's most challenging humanitarian contexts.

## Methods

### Procedure

The data report employed a cross-sectional quantitative design, focusing on individual participants as the unit of analysis. Eligibility criteria required participants to be 18 years of age or older. A self-selected sampling approach was adopted to the Google Forms platform. Simultaneously, we applied a convenience sampling method by engaging participants via various social media platforms (including Telegram FZ-LLC) and the official Facebook page of the Faculty of Psychology of Taras Shevchenko National University of Kyiv, aiming to reach a diverse demographic and regional representation across Ukraine. Data collection occurred in a single phase, spanning from December 10 to December 29, 2024. In total, 241 respondents met the inclusion criteria. Informed consent was collected from all participants. All data were processed in accordance with applicable data privacy regulations and ethical guidelines. All questions were presented in the Ukrainian language. As no Ukrainian adaptation was available for BRS, PCL-5, ISI, PSQI, PG-13, we used an author-translated version that underwent standard questionnaire translation procedures. During the data collection process, participants completed a series of validated psychological questionnaires, which are described below.

### Measurements

The Brief Resilience Scale (BRS) is a concise self-report measure designed to assess an individual's ability to recover or “bounce back” from stress. It includes six items scored on a Five-point Likert scale, ranging from 1 (“strongly disagree”) to 5 (“strongly agree”). The total score is calculated as the mean of all item responses, with reverse scoring applied to three negatively worded items. Higher scores indicate greater resilience (Smith et al., 2008).

The PHQ-9 is a short self-report questionnaire designed to screen for depression and assess its severity over the past 2 weeks. It consists of nine items corresponding to DSM-IV criteria for major depressive disorder. Each item is rated on a Four-point Likert scale from 0 (“not at all”) to 3 (“nearly every day”), with total scores ranging from 0 to 27. Scores are categorized as minimal (1–4), mild (5–9), moderate (10–14), moderately severe (15–19), or severe (20–27) depression (Kroenke et al., 2001). The PHQ-9 has been adapted for use in Ukrainian (Institute of Cognitive Behavioral Therapy, 2012).

The Posttraumatic Stress Disorder Checklist for DSM-5 (PCL-5) is a 20-item self-report questionnaire designed to evaluate PTSD symptoms based on DSM-5 criteria. Respondents rate each item on a Five-point Likert scale, ranging from 0 (“not at all”) to 4 (“extremely”), reflecting how much they have been bothered by PTSD symptoms over the past month. The PCL captures a broad range of posttraumatic symptoms that may emerge in response to stressors, even in the absence of a strong emotional identification with a specific traumatic experience. The total score ranges from 0 to 80, with higher scores indicating greater symptom severity (Karachevskii, 2016).

The GAD-7 is a brief self-report questionnaire that screens for generalized anxiety disorder (GAD) and measures its severity over the past 2 weeks. It contains seven items based on DSM-IV criteria for GAD, scored on a Four-point Likert scale from 0 (“not at all”) to 3 (“nearly every day”). Total scores range from 0 to 21, with thresholds of 5, 10, and 15 indicating mild, moderate, and severe anxiety, respectively. The GAD-7 is validated for use in both clinical and research settings (Aleksina et al., 2024). A cut-off score of 10 is commonly used to identify probable GAD cases, providing high sensitivity (89%) and specificity (82%). According to Williams (2014), a score of 10 or higher is also recommended as a threshold for referring individuals for further evaluation of anxiety disorders. This threshold was also applied in a similar sample by Lushchak et al. (2023).

The Insomnia Severity Index (ISI) is a self-report measure designed to assess the nature, severity, and impact of insomnia. It includes seven items evaluating sleep onset difficulties, sleep maintenance issues, early awakening, satisfaction with sleep patterns, interference with daily functioning, noticeability of sleep problems, and distress caused by sleep issues. Each item is scored on a Five-point Likert scale from 0 (“no problem”) to 4 (“very severe problem”), resulting in a total score ranging from 0 to 28. Scores are categorized as absence of insomnia (0–7), subthreshold insomnia (8–14), moderate insomnia (15–21), or severe insomnia (22–28). The ISI is widely validated for assessing both clinical severity and treatment outcomes (Bastien et al., 2001).

The Pittsburgh Sleep Quality Index (PSQI) is a self-report questionnaire designed to measure sleep quality and disturbances over the past month. It consists of 19 self-rated items that generate seven component scores: subjective sleep quality, sleep latency, sleep duration, habitual sleep efficiency, sleep disturbances, use of sleeping medication, and daytime dysfunction. These components are summed to produce a global score ranging from 0 to 21, with higher scores indicating poorer sleep quality. A global score above 5 suggests poor sleep quality (Buysse et al., 1989).

The WHO-5 Wellbeing Index is a short self-report questionnaire that measures subjective wellbeing. It consists of five items rated on a Six-point Likert scale from 0 (“never”) to 5 (“all the time”), reflecting the respondent's feelings over the past 2 weeks. The total score ranges from 0 to 25 and is converted into a percentage (0–100%) to represent overall wellbeing. Scores below 50 indicate poor wellbeing and warrant further evaluation for depression using ICD-10 criteria. The WHO-5 is widely used to monitor changes in wellbeing, with a 10% score change considered clinically significant. This tool has been adapted for use in Ukrainian (Karamushka et al., 2023; Topp et al., 2015).

The Continuous Traumatic Stress Response (CTSR) scale is a 15-item self-report questionnaire designed to measure symptoms of traumatic stress due to ongoing exposure to threats. Respondents rate their symptoms over the past month on a Four-point Likert scale from “not at all” to “to a very large extent.” The scale also evaluates distress and functional impairment using additional severity subscales. It includes items addressing prior trauma exposure and previous mental health diagnoses. The CTSR primarily assesses the subjective emotional experience and perceived severity of a traumatic event, focusing on the individual's internal sense of trauma. The CTSR was developed and validated to assess continuous traumatic stress in individuals facing ongoing security threats. This questionnaire has been adapted for use in Ukrainian (Frankova et al., 2025; Goral et al., 2021).

The Prolonged Grief Disorder (PG-13) scale is a diagnostic tool consisting of 13 items that evaluate symptoms of prolonged grief, including separation distress, cognitive and emotional symptoms, and functional impairment. Responses are scored using a Likert scale, ranging from “not at all” to “several times a day” or “overwhelmingly.” Specific diagnostic thresholds across five domains must be met to identify prolonged grief disorder (PGD). According to Prigerson et al. (2009), a diagnosis requires the presence of at least five out of nine specific symptoms, occurring either daily or to a disabling extent. These include emotional numbness, a sense of being stunned or feeling that life lacks meaning, mistrust, persistent bitterness about the loss, difficulty accepting the death, confusion about one's identity, avoidance of reminders of the loss, and an inability to move forward. These symptoms must persist for a minimum of 6 months following the death and must significantly impair daily functioning. The PG-13 was developed following evidence-based guidelines for diagnosing PGD (Prigerson et al., 2009).

The Alcohol Dependence Scale (ADS) includes 25 items designed to measure the severity of alcohol dependence. The questionnaire evaluates various aspects, including the quantity and frequency of alcohol use, physical and psychological symptoms, behavioral patterns, adverse effects, and attempts to control drinking. Responses are scored using frequency- and severity-based options, with some items employing a Likert-like format. The total score, calculated by summing item scores, provides an overall measure of dependence severity (Skinner and Horn, 1984).

### Data characteristics

The dataset comprises responses from 241 participants and includes demographic variables such as gender, age, marital status, education level (highest completed), monthly income (in UAH), and region of residence. The study presents findings on respondent distributions based on available cutoff scores (using either internationally established or previously published thresholds), along with descriptive statistics such as means and standard deviations. These are reported alongside reliability and validity indices for each questionnaire. Data preprocessing included scaling adjustments, such as multiplying WHO-5 scores by 4 (Topp et al., 2015) and dividing BRS-6 scores by 6 (Smith et al., 2008), to align with scoring conventions, ensuring accurate analysis and interpretation.

### Data cleaning

Responses from respondents under the age of 18 were excluded from the data collected. Respondents were required to answer all questions, thus yielding no missing values.

### Data description

In Table 1, we show gender, age, marital status, education, monthly income, and region of residence as the main demographic variables. Each category in a variable is analyzed using frequency and percentage.

**Table 1:** Overview of sociodemographic data.

**Variable**	**Category**	***N***	**%**
Gender	Female	191	79.3
	Male	47	19.5
	Other	3	1.2
Age	18–25 years	88	36.5
	26–35 years	69	28.6
	36–60 years	79	32.8
	61–75 years	5	2.1
Marital status	Widower/widow	2	0.8
	Civil marriage	29	12
	Single	119	49.4
	Married	70	29
	Divorced	17	7.1
	Other	4	1.7
Education	Bachelor's degree	63	26.1
	Master's degree	105	43.6
	I have a scientific degree	10	4.1
	Complete general secondary education (11 grades)	36	14.9
	Professional junior bachelor/junior specialist	18	7.6
	Other	9	3.8
Monthly income (UAH)	10,000–15,000	30	12.6
	15,000–20,000	33	13.9
	25,000–30,000	27	11.2
	5,000–10,000	21	8.7
	More than 30,000	73	30.7
	Less than 5,000	33	13.7
	I do not wish to answer	24	10
Region of residence	Ivano-Frankivsk	4	1.7
	Volyn	2	0.8
	Vinnytsia	3	1.2
	Dnipropetrovska	16	6.6
	Donetsk	1	0.4
	Zhytomyr	6	2.5
	Transcarpathian	1	0.4
	Zaporizhzhya	1	0.4
	Kyiv (together with the city of Kyiv)	115	47.7
	Kirovograd	5	2.1
	Lviv	16	6.6
	Mykolaiv	4	1.7
	Odesa	9	3.7
	Poltava	3	1.3
**Variable**	**Category**	***N***	**%**
	I do not live in Ukraine (abroad)	27	11.2
	Rivne	3	1.2
	Ternopil	1	0.4
	Kharkiv	9	3.7
	Kherson	1	0.4
	Khmelnytsky	4	1.7
	Cherkasy	4	1.7
	Chernivtsi	2	0.8
	Chernihiv	4	1.7

N, frequency; %, percentage.

In Table 2, we have presented Cronbach's alpha, composite reliability (CR), average variance extracted (AVE), tolerance, and variance inflation factor (VIF) as tools to assess the reliability and validity of the measures. All calculations were performed in R. Cronbach's alpha was computed using the psych package, and values above 0.7 were considered acceptable (Howard, 2021; Nasution et al., 2020). Composite reliability and average variance extracted were calculated using standardized item loadings from item correlation matrices, following standard psychometric formulas. Composite reliability should be at least 0.7 (Nasution et al., 2020). As for the AVE, it is a key indicator of convergent validity, which assesses whether a set of items truly reflects the underlying construct they are intended to measure. The generally accepted threshold for AVE is 0.50, meaning that the construct should explain at least 50% of the variance in its indicators (Shrestha, 2021). Ensuring AVE meets this level strengthens the evidence that the questionnaire reliably captures the intended theoretical concept. Tolerance and variance inflation factor (VIF) are important metrics for diagnosing multicollinearity among predictors in a regression model. Tolerance and VIF were calculated based on the AVE to screen for multicollinearity in the predictors. Tolerance, computed as 1–*R*^2^, indicates the proportion of variance in a predictor not explained by other predictors. Typically, a tolerance value below 0.10 signals problematic multicollinearity. VIF, the reciprocal of tolerance, quantifies the degree to which a predictor's variance is inflated due to multicollinearity. VIF helps detect if predictors are too highly correlated, which could distort regression coefficients and lead to unreliable conclusions. By monitoring VIF values, we can ensure that their models accurately reflect the distinct effects of different psychological constructs, and thus improve the validity and interpretability of their findings. Monitoring tolerance and VIF helps ensure reliable regression estimates (Kim, 2019). To ensure that multicollinearity did not bias the regression estimates, a threshold of VIF < 5 was adopted, consistent with established guidelines suggesting that VIF values above 5 warrant concern (Menard, 2001).

**Table 2:** Reliability and validity.

**Construct**	**Number of items**	**α**	**CR**	**AVE**	**Tolerance**	**VIF**
WHO-5	5	0.896	0.504	0.504	0.495	2.018
CTSR-15	15	0.907	0.194	0.194	0.806	1.241
PG-13	13	0.941	0.361	0.361	0.639	1.564
ADS	25	0.845	0.045	0.045	0.955	1.047
ISI-7	7	0.873	0.325	0.325	0.675	1.482
GAD-7	7	0.87	0.317	0.317	0.683	1.465
PCL-5	20	0.937	0.209	0.209	0.791	1.264
PHQ-9	9	0.88	0.262	0.262	0.738	1.355
BRS-6	6	0.879	0.388	0.388	0.612	1.634
PSQI	7	0.585	0.092	0.092	0.908	1.102

α, Cronbach's alpha; CR, composite reliability; AVE, average variance extracted; VIF, variance inflation factor; WHO-5, World Health Organization Wellbeing Index (5-item version); CTSR-15, Continuous Traumatic Stress Response scale (15-item version); PG-13, Prolonged Grief Disorder scale (13-item version); ADS, Alcohol Dependence scale; ISI-7, Insomnia Severity Index (7-item version); GAD-7, Generalized Anxiety Disorder scale (7-item version); PCL-5, posttraumatic stress disorder checklist for DSM-5 (20-item version); PHQ-9, patient health questionnaire (9-item version); BRS-6, Brief Resilience scale (6-item version); PSQI, Pittsburgh Sleep Quality Index.

Some constructs, including CTSR-15, ADS, and PSQI, showed low Average Variance Extracted (AVE) values (below the recommended threshold of 0.50), suggesting weak convergent validity. This indicates that these scales may not capture sufficient variance in their indicators within our sample, which could affect the reliability of related results. Consequently, caution is warranted when interpreting findings based on these measures, and future studies should further examine and refine these instruments to improve their validity and applicability.

To evaluate the structural validity of the questionnaires in our sample, we conducted a confirmatory factor analysis using the component scores, that is, the scores for each item, as observed variables. The CFA was performed using the “lavaan” package in R (version 4.4.1), employing the robust maximum likelihood (MLR) estimator. The results are presented in Table 3.

**Table 3:** Confirmatory factor analysis.

**Questionnaire**	**CFI**	**TLI**	**RMSEA**
ADS-25	0.421	0.368	0.144
BRS-6	0.991	0.985	0.052
CTSR-15	0.776	0.739	0.134
GAD-7	0.924	0.886	0.127
ISI-7	0.914	0.914	0.143
PCL-20	0.802	0.779	0.113
PG-13	0.804	0.765	0.178
PHQ-9	0.899	0.866	0.118
PSQI-7	0.924	0.886	0.059
WHO-5	0.968	0.935	0.139

CFI, Comparative Fit Index; TLI, Tucker-Lewis Index; RMSEA, Root mean square error of approximation.

As for the ADS-25, both indices (CFI and TLI) are low, and the RMSEA is higher than accepted, which indicates a poor model fit, suggesting that the one-factor model may not adequately capture the underlying structure of this questionnaire. In contrast, the BRS-6 demonstrated an accurate model fit, with CFI (0.991) and TLI (0.985) well above the conventional threshold of 0.95, and a low RMSEA (0.052), indicating a close fit to the data. The CTSR-15 showed a marginal fit, with CFI (0.776) and TLI (0.739) below the ideal cutoffs, and an RMSEA of 0.134, suggesting potential model misspecification. The GAD-7 and PSQI-7 both demonstrated acceptable model fit, with CFI values of 0.924 and RMSEA values close to or below 0.06. ISI-7 also showed a good fit, with CFI and TLI at 0.914 and an RMSEA slightly above 0.14, which may indicate a minor deviation from a perfect fit. The PCL-20 and PG-13 both exhibited suboptimal model fit, with CFI values just above 0.80 and TLI values below 0.78, along with relatively high RMSEA values (0.113 and 0.178, respectively). The PHQ-9 demonstrated a reasonable fit, with CFI (0.899) approaching acceptable levels and RMSEA (0.118) within a moderate range. Lastly, the WHO-5 showed a strong model fit, with a high CFI (0.968), TLI (0.935), and an SRMR below 0.03, although the RMSEA (0.139) was slightly elevated, suggesting room for improvement.

A potential explanation for the poor model fit in some scales is that the latent factor structure of a questionnaire may vary depending on the characteristics of the sample, such as cultural background, age, or psychological state. While minor modifications such as rewording items or recalibrating scoring could potentially improve model fit, such changes were not implemented in this study to preserve the original validated structure of the instruments and maintain comparability with previous research. Moreover, given the theoretical grounding and extensive prior use of these scales in trauma-related and cross-cultural contexts, they remain valuable tools for assessing psychological wellbeing.

Further research aimed at testing the measurement invariance of these instruments across different populations and contexts are needed for reliability and validity, as well as exploring alternative factor structures that may better reflect the specific features of the sample under investigation.

In Table 4, responses to each questionnaire are summarized. For Pittsburgh Sleep Quality Index (PSQI), we chose 5 as a cutoff value, based on previous studies (Buysse et al., 1989). Specifically, participants who scored >5 are considered to have poor sleep quality, and participants who scored 5 or less are considered to have good sleep quality. For ISI-7, differentiative thresholds are no insomnia, subthreshold insomnia, insomnia, moderate insomnia, and severe insomnia (Bastien et al., 2001). Concerning GAD-7, traditional levels are differentiated with scores 5, 10, and 15, creating mild, moderate, moderately severe, and severe anxiety cutoffs (Aleksina et al., 2024). For PCL-5, 41 was chosen as a cutoff score, meaning that respondents who scored more than 41 have symptoms of PTSD (Morrison et al., 2021). For PHQ-9, Concerning WHO-5, the cutoff score for the Ukrainian population is 50 (same as for Western Europe), so participants who scored 50 or lower are considered to have poor life quality, and those who scored more than 50—high life quality (Asanov et al., 2023). The BRS-6 questionnaire cutoffs are 3 and 4, meaning that scores below 3 indicate low resilience, and scores above 4.3 elicit high resilience (Smith et al., 2013). As for CTSR, the questionnaire consists of 15 items rated on a Four-point Likert scale ranging from 0 (“Not at all”) to 3 (“Often”). Total scores are obtained by summing the item scores, with higher scores indicating more severe symptoms of continuous traumatic stress. In line with previous research (Goral et al., 2021), a median score of 3 was used as a cutoff to distinguish between probable presence or absence of traumatic stress symptoms. For the PG-13 (Prolonged Grief Disorder) questionnaire, participants responded to 13 items rated on a Five-point Likert scale, assessing the frequency and intensity of grief-related symptoms. Total scores were calculated by summing relevant item responses. Participants were categorized into two groups—“No prolonged grief” and “Probable prolonged grief”—based on the diagnostic criteria outlined by Prigerson and Maciejewski (2006), which include symptom duration, functional impairment, and symptom severity.

**Table 4:** Summary of the results.

**Questionnaire**	**Symptom**	**Gender**	**Mean**	**SD**	**Cutoff**	**Count**	**Percentage**
PSQI	Sleep quality	Other	9	3.46	Poor sleep quality	3	100
		Female	6.48	2.93	Good sleep quality	57	29.84
					Poor sleep quality	134	70.16
		Male	6.26	2.65	Good sleep quality	15	31.91
					Poor sleep quality	32	68.09
ISI-7	Insomnia severity	Other	14	7	No insomnia	1	33.33
					Moderate insomnia	2	66.67
		Female	10.48	6.16	No insomnia	56	29.32
					Subthreshold insomnia	84	43.98
					Moderate insomnia	40	20.94
					Severe insomnia	11	5.76
		Male	9.94	5.92	No insomnia	18	38.3
					Subthreshold insomnia	16	34.04
					Moderate insomnia	10	21.28
					Severe insomnia	3	6.38
GAD-7	Anxiety	Other	13.67	5.13	Moderate anxiety	1	33.33
					Severe anxiety	2	66.67
		Female	9.21	4.95	Mild anxiety	39	20.42
					Moderate anxiety	68	35.6
					Moderately severe anxiety	52	27.23
					Severe anxiety	32	16.75
		Male	8.26	5.2	Mild anxiety	13	27.66
					Moderate anxiety	17	36.17
					Moderately severe anxiety	13	27.66
					Severe anxiety	4	8.51
PCL-5	PTSD	Other	47.67	24.09	No PTSD	1	33.33
					Probable PTSD	2	66.67
		Female	29.31	16.32	No PTSD	145	75.92
					Probable PTSD	46	24.08
		Male	29.3	17.33	No PTSD	36	76.6
					Probable PTSD	11	23.4
PHQ-9	Depression	Other	17.33	8.96	Mild depression	1	33.33
					Severe depression	2	66.67
		Female	11.24	5.9	Minimal depression	14	7.33
					Mild depression	59	30.89
					Moderate depression	53	27.75
					Moderately severe depression	38	19.9
					Severe depression	27	14.14
		Male	11.19	5.9	Minimal depression	5	10.64
					Mild depression	11	23.4
					Moderate depression	14	29.79
					Moderately severe depression	10	21.28
					Severe depression	7	14.89
WHO-5	Wellbeing	Other	25.33	26.63	Good wellbeing	2	66.67
					Poor wellbeing	1	33.33
		Female	41.88	20.89	Good wellbeing	126	65.97
					Poor wellbeing	65	34.03
		Male	44.77	25.4	Good wellbeing	27	57.45
					Poor wellbeing	20	42.55
BRS-6	Resilience	Other	1.61	0.59	Low resilience	3	100
		Female	2.84	0.84	Low resilience	102	53.4
					Normal resilience	80	41.88
					High resilience	9	4.71
		Male	3.04	0.93	Low resilience	19	40.43
					Normal resilience	23	48.94
					High resilience	5	10.64
CTSR-15	Trauma symptoms	Other	2.33	1.15	No significant trauma	1	33.33
					Probable trauma	2	66.67
		Female	0.99	0.86	No significant trauma	179	93.72
					Probable trauma	12	6.28
		Male	1.11	0.84	No significant trauma	44	93.62
					Probable trauma	3	6.38
PG-13	Prolonged grief	Other	31	16.5	No prolonged grief	3	100
		Female	18.9	9.5	No prolonged grief	186	97.38
			42.6	4.2	Prolonged grief	5	2.62
		Male	20.9	10.4	No prolonged grief	46	97.87
			46	NA	Prolonged grief	1	2.13
ADS	Alcohol dependence	Other	11.33	13.05	Low dependence	2	66.67
					Substantial dependence	1	33.33
		Female	3.03	3.4	Low dependence	188	98.43
					Intermediate dependence	3	1.57
		Male	4.89	5.73	Low dependence	43	91.49
					Intermediate dependence	3	6.38
					Substantial dependence	1	2.13

For the Alcohol Dependence Scale (ADS), participants completed 25 items measuring aspects of alcohol dependence. Scores were summed to produce a total score ranging from 0 to 47, with higher scores indicating greater alcohol dependence. Consistent with Murphy and MacKillop (2011), we used the following interpretation thresholds: 0–13 = low dependence, 14–21 = moderate dependence, 22–29 = substantial dependence, 30+ = severe dependence.

## Limitations

One limitation of the present study is the lack of a screening question to distinguish between civilian and military respondents. As a result, we cannot reliably separate civilian participants from those who may have been actively involved in military operations or affiliated with the armed forces.

Another limitation is related to the sampling strategy. Although the survey was distributed online to reach a diverse demographic and regional representation, recruitment was conducted mainly through a Telegram channel and the official Facebook page of the Faculty of Psychology at Taras Shevchenko National University of Kyiv. This approach may have limited the diversity of the sample in terms of age groups, socio-economic statuses, and regional backgrounds.

Moreover, not all cutoff scores used for the psychological questionnaires have been validated specifically for the Ukrainian population. In such cases, we relied on internationally established or widely accepted thresholds from prior research. While this allowed us to classify symptom severity and facilitate comparison with other studies, the cultural and contextual appropriateness of some cutoffs may be limited.

Some scales, such as the Alcohol Dependence Scale (ADS) and Pittsburgh Sleep Quality Index (PSQI), showed unexpectedly low composite reliability values in our sample. This suggests potential measurement limitations that should be addressed in future research. Certain questionnaires, such as CTSR-15, ADS, and PSQI, exhibited low AVE values, indicating weak convergent validity in our sample. This suggests that these instruments may not adequately capture the underlying constructs, potentially limiting the robustness of findings based on these measures. Future research should aim to further validate and possibly refine these scales within the Ukrainian population to enhance their psychometric properties and interpretability.

## Data Availability

The datasets presented in this study can be found in online repositories. The names of the repository/repositories and accession number(s) can be found below: https://osf.io/smhnr/.
